# Modeling aquifer storage and recovery in the eastern district of the United Arab Emirates using MODFLOW

**DOI:** 10.1038/s41598-022-20470-7

**Published:** 2022-10-20

**Authors:** Karim Khalil, Qasim Khan, Mohamed Mohamed

**Affiliations:** 1 Civil & Environmental Engineering Department, United Arab Emirates University, 15551 Al Ain, UAE; 2 National Water and Energy Center, United Arab Emirates University, 15551 Al Ain, UAE

**Keywords:** Hydrology, Environmental sciences

## Abstract

The Emirate of Abu Dhabi has relied on groundwater as a source of fresh water for several decades, which has resulted in the deterioration of non-renewable groundwater aquifers. This has led to the installation of desalination plants for fresh water supply. This research aims to increase strategic water reserves in the eastern district of Abu Dhabi by analyzing the best locations for aquifer storage and recovery (ASR). The ASR technology offers an opportunity to store large volumes of water for later beneficial use. This study explores an option of using excess desalination water for ASR recharge in the eastern district Al Ain region of Abu Dhabi. A limiting factor in the application of the ASR technology is the lack of suitable sites. Detailed hydrogeological and operational knowledge of the studied areas helped in identifying potential sites for ASR based on a scoring system. Determining best locations for managed aquifer recharge is a crucial design step. Five scenarios were studied at Al-Khrair and Al-Shuwaib sites in Al Ain region. Results show that a wider distribution of injection wells with intervals more than 1200 m is more suitable to overcome the excessive head buildup. Based on the adopted criteria, Al-Khrair was the best site for recharge followed by Al-Shuwaib. Al-Khrair site can be recharged at 64,000 m^3^ d^−1^ for seven years, while Al-Shuwaib site can be recharged at 64,000 m^3^ d^−1^ for only two years.

## Introduction

The supply of freshwater is a global challenge that gives rise to issues related to water security^1–4^. The rapid development and continuous growth of the population in the United Arab Emirates (UAE) has resulted in an increase in water demand^5,6^. According to the Statistical Yearbook of Abu Dhabi (SCAD)^7^, the population of the Al Ain region was 738,500 in mid-2015, with an increase of around 26% compared to the population in mid-2010. According to Younis^8^, the population of the Al Ain region is expected to be doubled in 2030 to reach 1,373,265. Abu Dhabi City has a per capita water consumption of 590 L d^−1^ according to the Environment Agency, Abu Dhabi^9^. Challenges to maintain a sustainable water supply include the absence of surface water, due to scarcity of rainfall, and high evaporation levels; thus, groundwater is the only conventional water resource^10^. In addition to population growth, other factors such as the expansion of irrigated agricultural lands also require groundwater. The consumption of groundwater by agricultural activities, households, and other uses are 90, 2, and 8%, respectively. The annual groundwater recharge and abstraction in the UAE were estimated to be 120 and 880 Mm^3^ yr^−1^, respectively^11^. A large portion of the water demand is provided by desalinated water produced by coastal desalination plants such as the Taweela desalination plant, which employs multi-stage flash (MSF) and multi-effect distillation (MED) technology, in the Emirate of Abu Dhabi and the Jabal Ali desalination plant, which operates on the MSF technology, in the Emirate of Dubai^12^. In the Emirate of Abu Dhabi, 71.3% of the desalinated water is consumed by agriculture, forestry, and landscaping, which is estimated to be more than 2,000 Mm^3^ yr^−1^, 16.5% by the domestic sector, 4.7% by the governmental sector, 6.5% by the commercial sector, 0.8% by the industrial sector, and 0.1% by other sectors^9^ water demand^13^.

The need for an alternative approach to manage water demand and provide uninterrupted freshwater supply is a major concern in the Emirate of Abu Dhabi^14^. Managed aquifer recharge is considered a cost-effective technique compared to aboveground alternatives that require the construction of water treatment plants, surface reservoirs, and large tracts of land. In addition, there may be insufficient space for aboveground water storage tanks, especially in urban areas^13,15^. Therefore, the Emirate of Abu Dhabi needs a large storage system that will overcome water demand during peak periods (from June to August), emergencies, and periods when desalination plants are out of commission for reasons such as natural disasters, industrial accidents, war, oil spills, and other crises.

Aquifer storage and recovery (ASR) is a water storage and treatment technology that was developed in the United States in 1968 and it first began operating in Wildwood, New Jersey^16^. ASR, through a system of groundwater wells, stores water underground through one or more wells and the water is recovered from the same well/wells later for supply^16–18^. The largest ASR wellfield is in Las Vegas, Nevada and it has more than 500,000 m^3^ d^−1^ of recovery capacity^16^. “ASR is favored by many countries because there are insignificant evaporation losses, and the stored water is less vulnerable to contamination by animals or humans”.

Many Gulf countries have studied the behavior of groundwater flow under specific recharge rates using injection wells. The most recent ASR system is in Liwa, United Arab Emirates. The ASR plant construction began in 2009 and it was completed in 2016. The infiltration of desalinated seawater began in 2015^19^ and the project aims to store a surplus of 23 Mm^3^ of desalinated water into an aquifer^20^. The reason for the occurrence of excess desalinated water is because most of the desalination plants in UAE are coupled with powerplants^21^. The unique composition of desalinated water compared to natural groundwater presents scientific and operational challenges that require further research^22^ such as clogging and compatibility, which are the main concerns in clastic aquifers^23^.

Understanding the local hydrogeological settings and groundwater modeling requires a comprehensive conceptual model and data on aquifer hydraulics, which are the main challenges in the prediction of the performance of an ASR system^24–28^. Inadequate planning and improper ASR site characterization and optimization are the main causes of the failure of an ASR system^15^. The salinity of the native groundwater should be determined as it affects the recovery efficiency of the ASR system because of the mixing of injected water with native saline groundwater, forming a bubble that drifts upward owing to differences in density. In addition, regional groundwater flow should be considered to avoid the possibility of the migration or loss of injected water during recovery as a result of the lateral bubble drift^18^. The formed bubble can be monitored over time if well-designed geophysical programs, such as surface electrical resistivity and borehole electrical tomography techniques, are established in the site area^15,24^. Furthermore, adverse environmental impacts, such as contamination of the groundwater, changes in the groundwater level, or unwanted changes in the saltwater–freshwater interface, can occur because of the construction of the ASR system^15,29^. Therefore, a full environmental impact assessment study is required before the construction of any ASR system^30,31^.

Furthermore, the injection rate and the density of distribution of injection wells are important in the implementation of an ASR at a specific site. High injection rate or closely spaced injection wells can cause head buildup, a process causes water table to rise above ground surface in low lands^32^. Excessive head buildup can cause groundwater management issues such as anthropogenic contamination, specifically in areas with a higher population density^33,34^. Additionally, contaminants presents on the surface, such as fertilizers in irrigated lands, can contribute to the contamination of the groundwater^35^.

In the Emirate of Abu Dhabi, the Environment Agency of Abu Dhabi (EAD), Abu Dhabi Water and Electricity Authority (ADWEA), German Technical Cooperation (GTZ), and United States Geological Survey (USGS) have conducted several studies. These studies simulated three model scenarios to allow the construction of ASR pilot projects in the Al Ain region^36^. The model scenarios were based on simulating a recharge of 1,000 m^3^ d^−1^ through scenarios of one injection well and two infiltration ponds using Visual Flex MODFLOW software to analyze the capacity of the aquifer to store water. The analyzed preliminary stage models were cost effective and demonstrated the feasibility of implementing an ASR system in Al Ain region to create a strategic freshwater reserve and replenish the depleted aquifer.

This paper is a continuation of previous efforts aiming to model different scenarios of injection rates and injection well distributions at the Al-Shuwaib and Al-Khrair sites in Al Ain region. A site-scoring procedure was implemented for 20 available discrete sites to select two ASR sites based on site-specific hydrogeological criteria and additional components. Then, a 3D groundwater model was developed using MODFLOW software. The model was calibrated using available data. The calibrated model was, then, used to analyze the performance of the selected ASR sites, using a finer model grid in the study locations, to simulate the capacity of the ASR system to recharge 64,000 m^3^ d^−1^ of water from 2013 to 2030. The recharge process was simulated using different numbers of injection wells and two well-distribution types to examine the ASR system performance in various situations. The results of this study will help authorities plan, develop, and manage groundwater resources in the region.

## Materials and methods

### Study area

Al Ain is located in the eastern region of the Emirate of Abu Dhabi in the UAE. The Emirate of Abu Dhabi covers 67,340 km^2^ and it is located in the southeast of the Arabian Peninsula. The region has a dry climate and is categorized as a hyperarid region. The temperature is usually 18 °C during December–February and 50 °C during June–August^25^. The study area is characterized by extremely low precipitation (100 mm yr^−1^), which is comparatively higher than the western region of Abu Dhabi due to the presence of mountains^37^. Al Ain region has fresh groundwater underflow through alluvial sediments in wadis drained from the Omani Mountains (Al-Hajar Mountains) and it experiences periodic storm runoff from water concentrated in wadis as compared to the rest of the country^38^.

**Figure 1:**
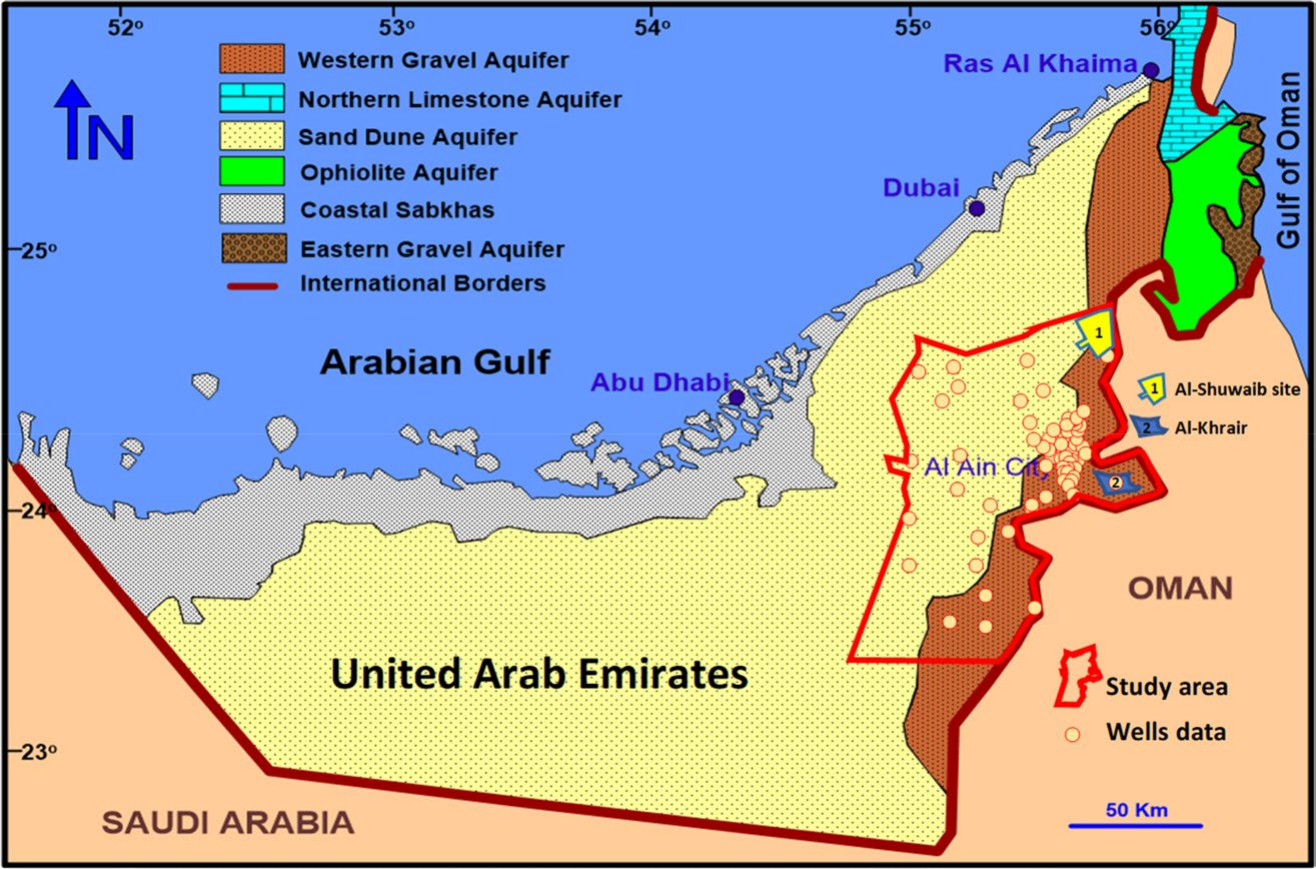
Map of the United Arab Emirates showing the study area (Al Ain region), the hydrological aquifers, and the two selected sites (Al-Shuwaib and Al-Khrair) (Modified after Rizk and Alsharan^39^ using ArcMap 10.8 https://www.esri.com/).

There are four main aquifers in the UAE^39^, the limestone aquifer in the northeast, gravel aquifer in the east (study area), ophiolite aquifer in the northeast, and sand dune aquifer in the south and west, as shown in Fig. 1.

Three geological layers were previously reported in Al Ain by Hutchinson^36^ and Brook^40^: surficial aquifer (sand and gravel aquifer), upper Fars Formation, and lower Fars Formation. Boreholes drilled by the National Drilling Company (NDC) and USGS during the 1990s in Al Ain showed a hydrogeological connection between the bottom of the surficial aquifer and the underlying upper Fars Formation in the eastern part of the Oman Mountains^41^. Currently, groundwater is regularly pumped from the upper Fars Formation, which has significantly reduced the groundwater table by 40–60 m from its historical elevation^25^. Thus, in this study, the surficial aquifer layer (unconfined and highly productive quaternary alluvium) and the upper Fars Formation are conceptualized as one layer overlying the lower Fars Formation. The lower Fars Formation is considered the bottom of the aquifer, which is on top of the confining layer (impermeable layer), as shown in Table 1. Hence, groundwater recharge was modeled in an unconfined aquifer.

**Table 1:**
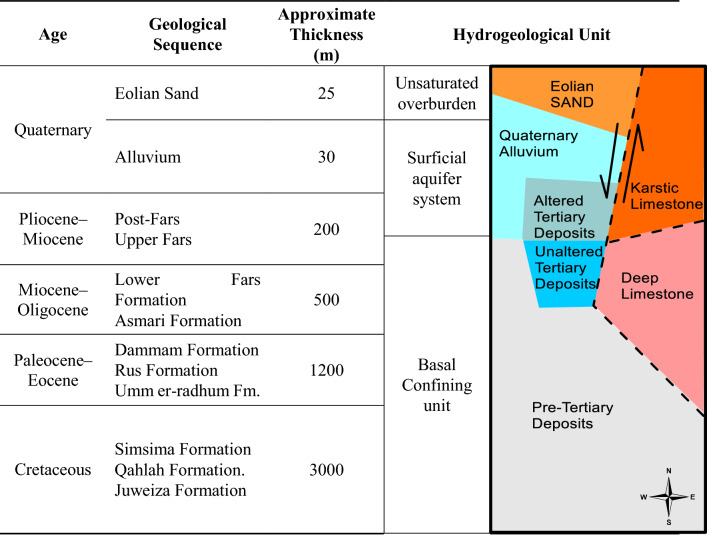
Hydrogeological framework of the eastern part of Abu Dhabi Emirate (Hutchinson, 1998).

### ASR site selection and scoring process

Data required for the potential ASR suitability assessment were obtained from the Arab Center for Engineering Studies Company (ACES) for a specified period from 2013 to 2017. Well data includes groundwater well coordinates, ground elevation in meters above sea level, depth of the aquifer, groundwater level, and lithology. In addition, data regarding the specific yield, permeability/transmissivity, and lithology were collected from the National Drilling Company (NDC) ^42^. The collected well log data were imported into a Microsoft Excel spreadsheet for well selection. The well selection process was implemented to examine the coordinates, elevation, depth, recorded groundwater level, and lithology. Twenty discrete sites at different locations within Al Ain region were identified as having the required information for the evaluation. Two groups of criteria were considered for the ASR site selection, including hydrological components (aquifer thickness, depth to water level, aquifer confinement, uniformity of hydraulic properties, groundwater salinity, hydraulic gradient and consolidation) and complementary components (aquifer minerology, redox state of native groundwater, permeability, well density within a radius of 1 km, recharge water quality, distance to source water, endangered species and predicted water supply) were adopted from Khalil et al.^43^. A score was assigned to each criterion based on its suitability. A score of 1 was assigned for poor/unsuitable, 2 for fair/limited suitability, 3 for good/suitable, and 4 for excellent/highly suitable^43^.

The score of each criterion of the hydrogeological and the complementary components is multiplied by a weighting factor. The weighting factor of each criterion is based on its impact on the efficiency of ASR^44,45^. The calculated scores were summed to obtain the total score for each site and used to create final suitability maps to rank the sites^44–47^. Results of the site scoring process indicated that the highest suitability scores (out of a maximum possible score of 160) were obtained at Al-Khrair and Al-Shuwaib sites with 89% and 81%, respectively (Table 2). Accordingly, these sites were selected for modelling in this paper.

**Table 2:** Aquifer storage and recovery (ASR) scores for Al-Shuwaib and Al-Khrair by applying the weighting factor.

Characteristics	Weighting factor	Sites			
		Al-Shuwaib		Al-Khrair	
		Score	Total score	Score	Total score
Thickness of the aquifer (m)	4	3	12	3	12
Permeability (m s^−1^)	4	4	16	4	16
Aquifer confinement	3	3	9	3	9
Uniformity of hydraulic properties	3	2	12	4	12
Groundwater salinity	4	4	16	4	16
Hydraulic gradient	3	2	6	2	6
Consolidation (porosity)	2	4	8	3	6
Aquifer mineralogy	1	4	4	4	4
Redox state of native groundwater	1	1	1	4	4
Depth to water level (m)	3	3	9	4	12
Well density	2	3	6	4	8
Recharge water quality	2	4	8	4	8
Distance to Source Water	3	3	9	3	9
Endangered species	2	4	8	4	8
Predicted water supply exceeds demand	3	4	12	4	12
Total score			136		142

The modeling simulations of the two selected ASR sites were conducted to assess possibility to store water without significant impacts on groundwater behavior or excessive head buildup. The assessment was based on different scenarios of injection rates with different numbers of injection wells and distributions.

### Model development

Visual MODFLOW Flex 2015.1 software (VMOD Flex) employs finite-difference method to solve the following three-dimensional equation that describes the movement of groundwater through porous earth media (Eq. 1^48)^:

1 $$\frac{\partial }{\partial x}\left( {K_{{{\text{xx}}}} \frac{\partial h}{{\partial x}}} \right) + \frac{\partial }{\partial y}\left( {K_{{{\text{yy}}}} \frac{\partial h}{{\partial y}}} \right) + \frac{\partial }{\partial z}\left( {K_{{{\text{zz}}}} \frac{\partial h}{{\partial z}}} \right) - W = { }S_{{\text{s}}} \frac{\partial h}{{\partial t}}$$

where ${K}_{\mathrm{xx}}$,${K}_{\mathrm{yy}}$, and ${K}_{\mathrm{zz}}$ are the hydraulic conductivities along the x-, y-, and z-axes, respectively, which are assumed to be parallel to the major axes of hydraulic conductivity (LT^−1^); $h$ is the groundwater head (L); $W$ is the volumetric flux per unit volume and represents sources and/or sinks of water (T^−1^); ${S}_{s}$ is the specific storage of the porous media (L^−1^); and t is the time (T). The first step prior to simulation was to build a conceptual model of the groundwater system. The property zones (assigning property values for conductivity, storage, and initial heads) and boundary conditions for each active grid cell used in the model.

### Data preparation

The top of the aquifer was represented by topographic data (ground surface), and the bottom of the aquifer/top of the confining layer was obtained from various borehole data in the study area obtained from ACES and NDC^42^. The ordinary kriging method was employed to interpolate the bottom of the aquifer layer. Values of hydraulic conductivity (m/s), specific storage (1/m), and initial head (m), were assigned to each cell of the model. Horizontal and vertical hydraulic conductivities were assigned based on data collected from Al-Shahi^42^. Forty-five data points of horizontal hydraulic conductivity were used within the study area with a maximum value of 0.003 m/s, minimum value of 1.157E-6 m/s, and average value of 0.0002 m/s, while the vertical hydraulic conductivity was assigned to be tenth of the horizontal conductivity^41^.

Several model calibration runs with different reported values of specific yield, total porosity, effective porosity, and specific storage were conducted until a good match was achieved between the observed and calculated hydraulic head values at twenty observation wells. According to several reports and tests conducted in the study area, the specific yield measured ranges from 0.01 to 0.27^42^, while it ranges from 0.02 to 0.18 in the alluvium aquifer^49^ with an average of 0.14^50^. Sathish and Mohamed^25^ used specific yields ranging from 0.01 to 0.32 in the eastern district of the UAE.

The aquifer in the study area had a high porosity of 0.4^51^, indicating potential for ASR^52^. However, a sensitivity analysis was performed through several trials using aquifer parameters such as specific yield, specific storage, effective porosity and total porosity to achieve the best results and the uncertainties in them were removed.

The developed model was used to simulate several runs using uniform grids with different sizes until the average difference “error” between values of measured and modeled hydraulic heads at the twenty observation wells is less than 0.5 m. The average error lowest value was 0.24 for a grid size of 100 × 100. Thus, the study area was modeled using finite difference grid of 100 rows and 100 columns (matrix of 10,000 grid cells). The dimensions of each cell were 1,169.6 m in width and 1,556.7 m in length. The entire model domain area was 18,207 km^2^ and the study area was approximately 13,000 km^2^.

### Boundary conditions

**Figure 2:**
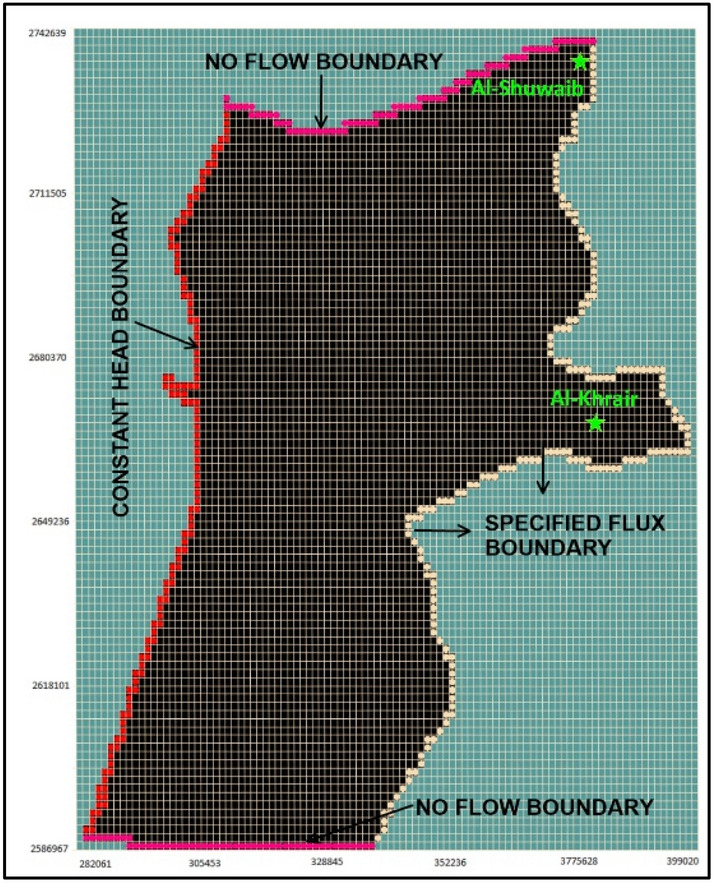
Boundary conditions used in the model.

Three types of boundary conditions were used in the study model: no flow, constant head, and specified flux as shown in Fig. 2. The regional groundwater flow direction is from the eastern boundary of the study area toward the West Arabian Gulf. The northern and southern boundaries of the study area were assigned as no-flow boundaries.

A constant head boundary was used at west of the study area. Transient specified flux was used at the eastern boundary of the study area to represent the recharge to the groundwater flow system from the Omani Mountain and wadis. The transient specific fluxes from the eastern boundary of the model were assigned based on the catchment flow to the Emirate of Abu Dhabi^40^, which is estimated at 30.9 Mm^3^ yr^−1^ of water recharge from the Omani Mountains that flows from twelve catchments bounded by the eastern boundary of the study area at various intensities. A constant head of 110 m was implemented at the western part of the study area as obtained from the Environmental Atlas of Abu Dhabi Emirate^53^.

### Model calibration and sensitivity analysis

The purpose of model calibration is to create a reliable groundwater model^54^. In this paper, the trial and error calibration technique was implemented. This technique is still favorable in several cases over the automated calibration known as parameter estimation^55^. Visual Flex MODFLOW was set to run from January 1, 2013. Observation well data distributed all over the model domain obtained from the ACES enabled the calibration of 18 stress periods from 2013 to 2017^56^.

**Figure 3:**
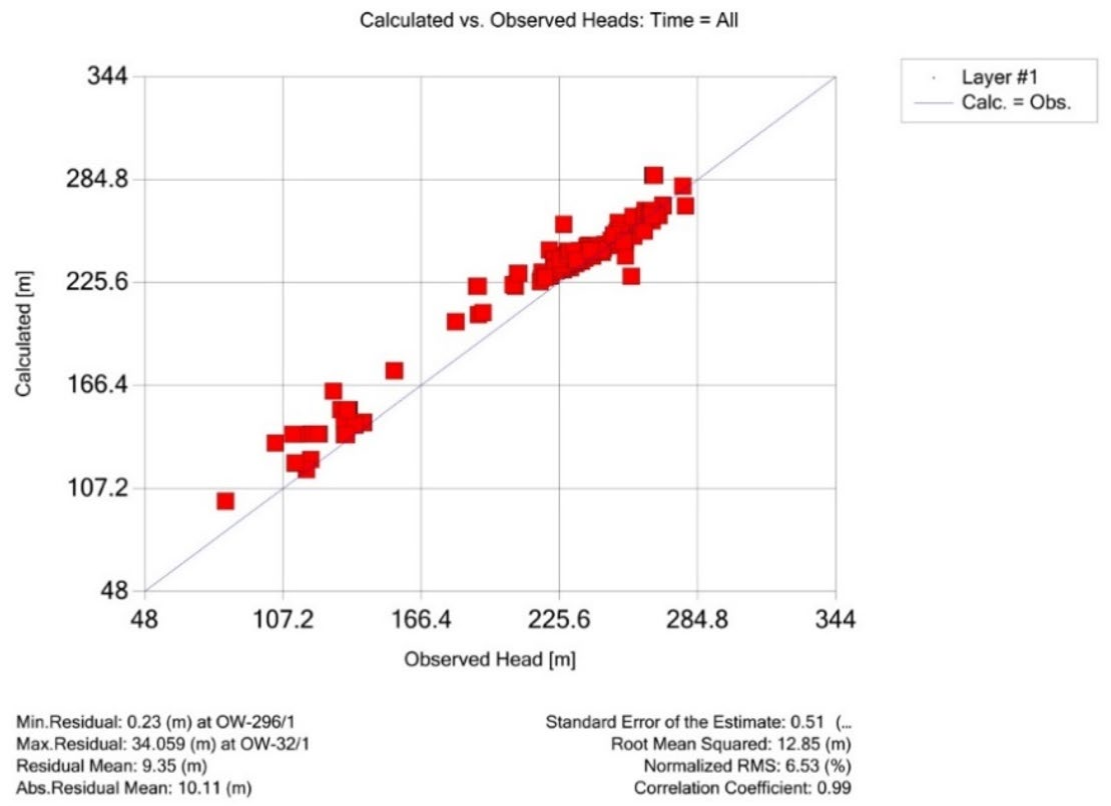
Visual MODFLOW (VMOD) output calibration chart comparing simulated and observed groundwater levels for all observation wells from 2013 to 2017.

After several trial-and-error runs and adjustments of aquifer parameters to reduce the difference between the observed and calculated hydraulic heads, the calibrated aquifer parameters used in the model had a uniform specific yield of 0.14, total porosity of 0.4, effective porosity of 0.25, and specific storage of 0.009 (1/m). The validation of the model was conducted, after the transient calibration, by comparing the calculated and observed groundwater levels at all observation wells from 2013 to 2017 (Appendix A). The obtained correlation coefficient was 0.99 (Fig. 3), which indicates that the simulated groundwater levels by the model were in good agreement with the observed values in the filed. The standard error was 0.51, while the Root-mean-square error and normalized root mean square error were 12.85 m and 6.53%, respectively. These errors are expected to be reduced even further if the groundwater pumping for desert greenery activities is considered in the model. Impact of desert greenery, which consists of man-made forests and greenhouse farming, was not considered due to the high uncertainties in the collected data (from 2013)^41^.

A global sensitivity analysis using a factorial analysis technique was implemented. The analysis showed that changes in groundwater levels were sensitive to changes in specific storage and specific yield. Based in this analysis, the average specific yield and specific storage were chosen as 0.14 and 0.009. These values are in agreement with values reported in literature for the study area^42,50^.

## Results

**Figure 4:**
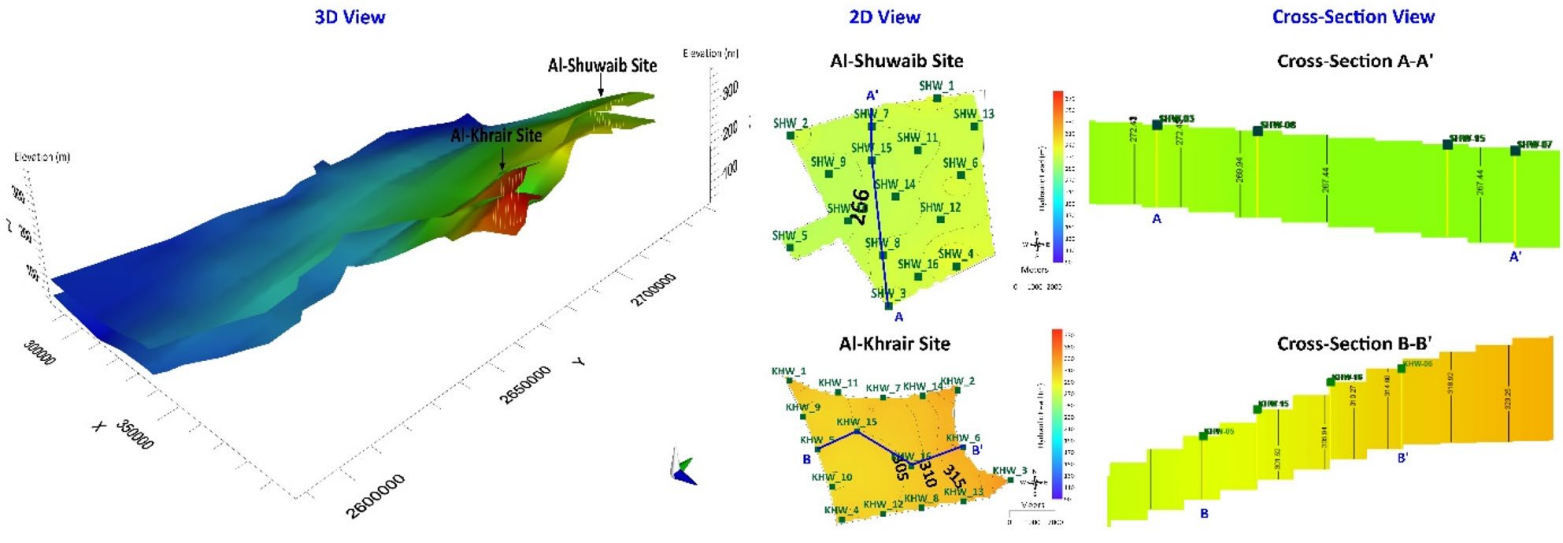
2D cross-section profile of Al-Shuwaib and Al-Khrair sites showing ASR wells.

The calibrated model was used to evaluate Al-Shuwaib and Al-Khrair sites in the study area. The eastern boundary of the model was assigned a specific flux boundary condition owing to the periodic recharge from the Omani Mountains. The transmissivity of the aquifer, well hydraulics, clogging rate, and availability of water are the main factors that determine the rate of injection^23^. The selected sites were simulated using five water-injection scenarios. The aim of each scenario was to simulate and understand the groundwater flow behavior under various recharge rates from injection wells^57^. The aquifer thicknesses at Al-Shuwaib and Al-Khrair are approximately 20 and 40 m, respectively. The cross-sectional profiles of these two sites along with the 3D view of the topography and bottom of the aquifer are shown in Fig. 4. The two sites are located at a higher elevation compared to other locations in the west and south.

The ASR scenarios aim to recharge Al-Shuwaib and Al-Khrair sites with a 64,000 m^3^ d^−1^ surplus from desalination plants in Abu Dhabi^20^ for the purpose of storage as a strategic water reserve. The recharge was simulated through multiple injection wells distributed over the selected sites rather than a single well to overcome the excessive head buildup at the water injection location. Recharge values of 32,000 m^3^ d^−1^ and 16,000 m^3^ d^−1^ were simulated to determine the best site for the ASR system and its storage capabilities.

### Distribution of injection wells

Five scenarios were developed with three main water recharge rates of 16000 m^3^ d^−1^, 32000 m^3^ d^−1^, and 64000 m^3^ d^−1^, using multiple injection wells to simulate the hydraulic head at the Al-Khrair and Al-Shuwaib sites, as listed in Table 3. The distribution of injection wells was considered in the assessment of the Al-Khrair and Al-Shuwaib sites. The model was simulated from 2013 to 2030 with water recharge rates of 1,000 m^3^ d^−1^ and 4000 m^3^ d^−1^ through 16 injection wells (Scenarios 1 and 5), 4000 m^3^ d^−1^ and 8000 m^3^ d^−1^ through eight injection wells (Scenarios 3 and 4), and 4,000 m^3^ d^−1^ through four injection wells (Scenario 2) located within the boundaries of Al-Khrair and Al-Shuwaib sites.

**Table 3:** Simulated injection scenarios.

Scenario	Number of injection wells per site	Recharge rate (m^3^ d^−1^)	Total recharge rate (m^3^ d^−1^)
1	16	1000	16,000
2	4	8000	32,000
3	8	4000	32,000
4	8	8000	64,000
5	16	4000	64,000

#### Al-Shuwaib site

**Figure 5:**
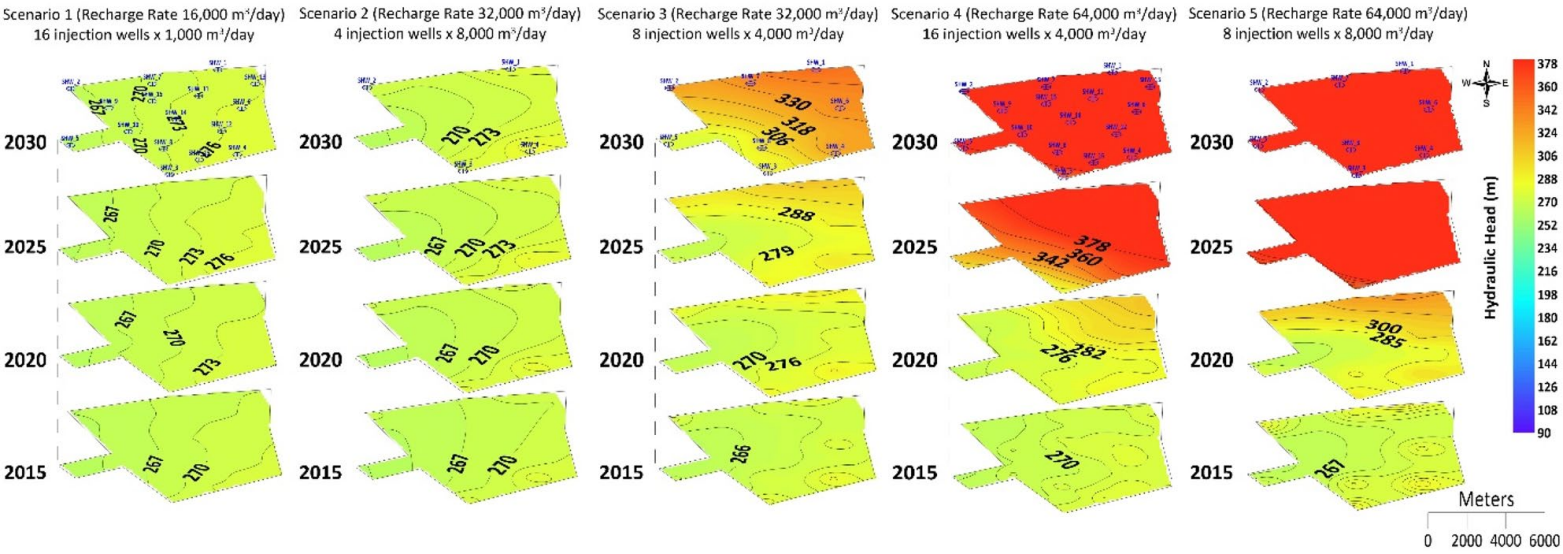
Simulated hydraulic heads at Al-Shuwaib site for the five recharge scenarios.

Results for the Al-Shuwaib site show that the hydraulic head increased in Scenario 1 from 262 m in 2015 to 267 m in 2030 at the western part of the site, while at the eastern part of the site it increased from 272 m in 2015 to 277 m in 2030 (Fig. 5). For Scenario 2, the hydraulic head increased slightly from 2015 to 2030 from 265 to 274 m, flowing toward the west of the site. A minor formation of a groundwater mound was observed surrounding four injection wells in 2025, and an increase in the hydraulic head from 270 m in 2015 to 274 m in 2030 was also observed in the eastern part of the site. However, for Scenario 3, the hydraulic head in 2015 was 274 m in the vicinity of SHW-1, SHW-3, and SHW-4 and it increased to 350 m by 2030, forming a groundwater mound. The minimum hydraulic head was 261 m at SHW-05 and it increased to 279 m by 2030. For Scenario 4, the hydraulic head increased from 265 m in 2015 to 400 m in 2030 in the western part of the site and it increased from 277 m in 2015 to 400 m in 2030 to the east of the site. For Scenario 5, the hydraulic head was 277 m at SHW-01 to SHW-04 and it was 262 m at SHW-05 in 2015. The values of the hydraulic head increased continuously until 2020 to 382 m at SHW-01, 302 m at SHW-02, 282 m at SHW-03, 292 m at SHW-04, and 267 m at SHW-05. In 2025, most of the sites had a hydraulic head > 380 m, except at SHW-05, which had 362 m. In 2030, the hydraulic head exceeded 380 m at all locations of the site.

#### Al-Khrair site

**Figure 6:**
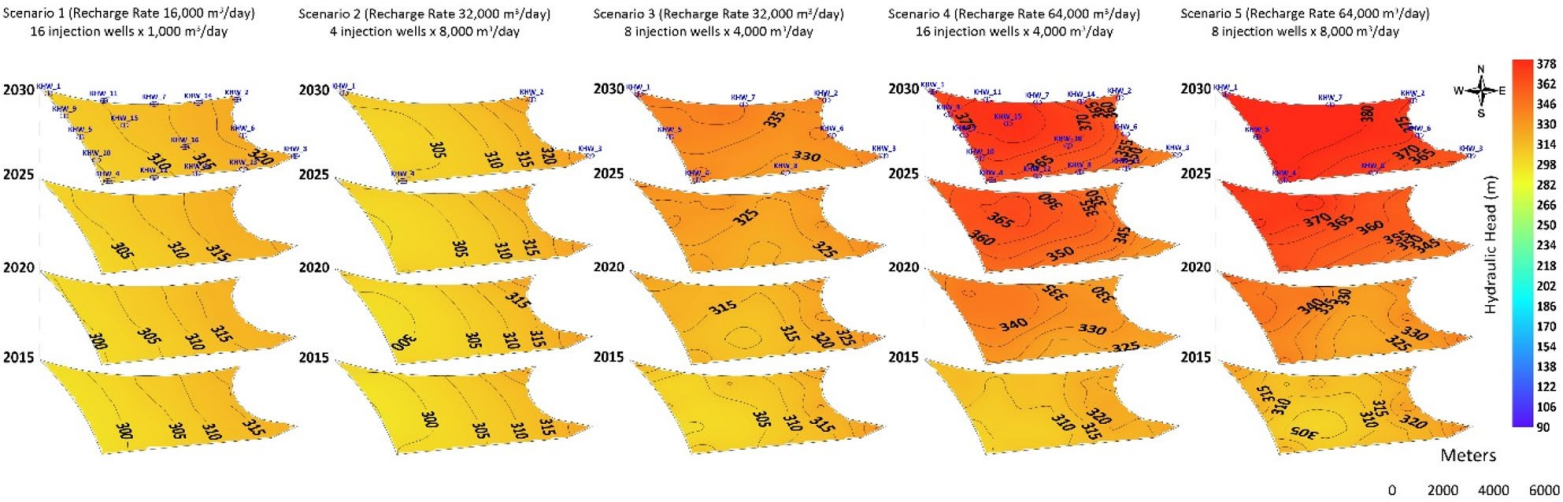
Simulated hydraulic heads at Al-Khrair site for the five recharge scenarios.

Results of Scenario 1 for the Al-Khrair site show that the hydraulic head increased from 297 m in 2015 to 307 m in 2030 in the western part of the site, and at the eastern part of the site it increased from 317 m in 2015 to 320 m in 2030 (Fig. 6).

For four injection wells with a recharge rate of 32,000 m^3^ d^−1 ^(Scenario 2), the hydraulic head in the Al-Khrair site increased from 298 m in 2015 to 302 m in 2030 in the western part of the site, and it increased from 316 m in 2015 to 321 m in 2030 in the eastern part of the site. A slight change in the hydraulic head was observed at the four injection wells located in the corners of the site boundary (KHW-1, KHW-2, KHW-3, and KHW-4) by 2030. However, for Scenario 3 (eight injection wells with a recharge rate of 32,000 m^3^ d^−1^), the hydraulic head increased significantly from 307 m in 2015 to 340 m in 2030 in the western part, while it increased from 320 m in 2015 to 332 m in 2030 in the eastern part of the site. A local groundwater mound formed around KHW-1, KHW-5, and KHW-6 in 2015. In 2020, the groundwater mound was formed around KHW-1, KHW-4, KHW-5, and KHW-8, while the hydraulic head increased at KHW-6. In 2025, changes in the hydraulic head were observed, and groundwater mounds formed toward the northwestern part of the site with a hydraulic head of 330 m and it reached 340 m in 2030. For 16 injection wells with a recharge rate of 64,000 m^3^ d^−1^(Scenario 5), the hydraulic head increased from 307 m in 2015 at the western part of the site to 367 m in 2030, and in the eastern part of the site it increased from 322 m in 2015 to 356 m in 2030. An isolated reverse cone of depression developed around KHW-1 in 2015 and it started to increase with time, forming a larger groundwater mound around injection wells KHW-11 and KHW-15 in 2030. However, for Scenario 4 (eight injection wells with a recharge rate of 64,000 m^3^ d^−1^), the hydraulic head formed an isolated reverse cone of depression around injection wells KHW-01, KHW-02, KHW-03, and KHW-04 in 2015. Hydraulic heads of 317 m at KHW-01, 327 m at KHW-02 and KHW-03, and 307 m at KHW-04 were observed in 2015. These values increased in 2020 to 347 m in KHW-1, 332 m in KHW-02 and KHW-03, and 332 m in KHW-04. In the northwestern part of the site, a groundwater mound developed at injection well KHW-01 with a hydraulic head of 387 m in 2030.

### Comparison between ASR scenarios

A comparison of the simulated hydraulic head in 2030 is presented to determine the most suitable site for implementation of an ASR system. This comparison will help in identifying the least sensitive site to significant increases in hydraulic head and changes in groundwater flow.

**Figure 7:**
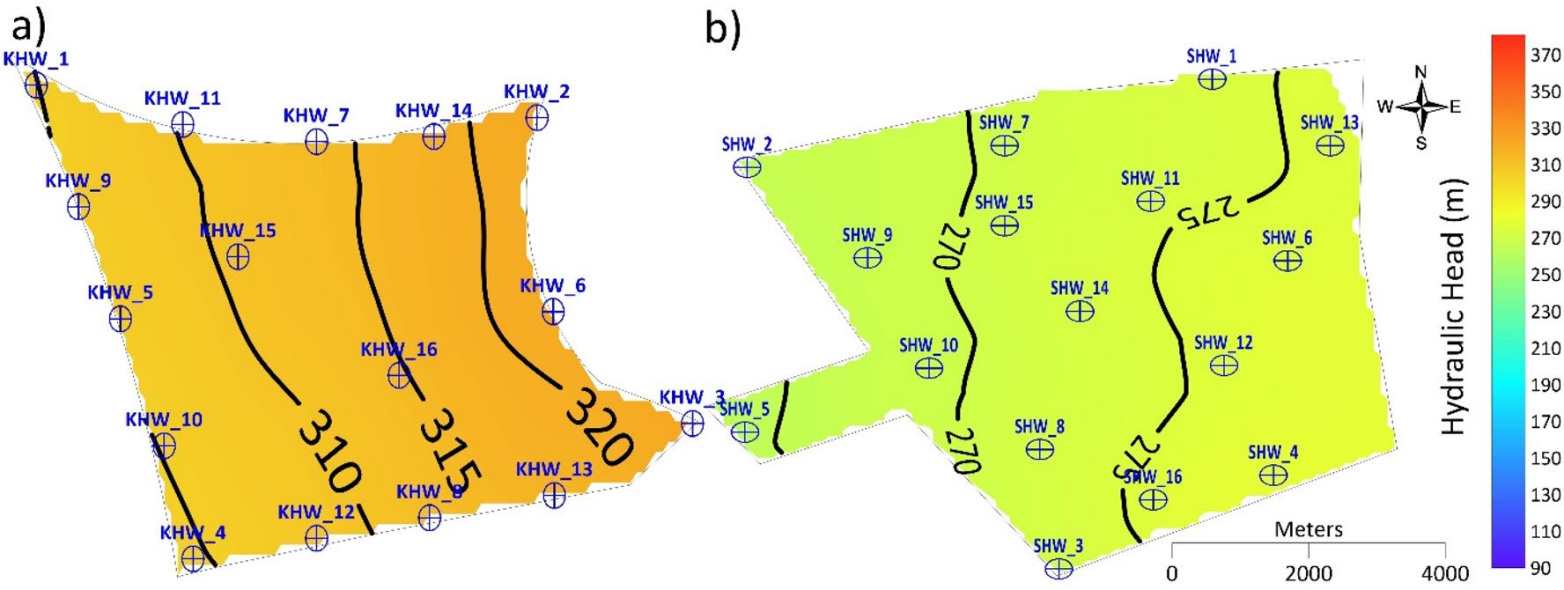
Simulated hydraulic head in 2030 for Scenario 1 at (**a**) Al-Khrair and (**b**) Al-Shuwaib sites.

The values of hydraulic head obtained at the site after a total recharge rate of 16,000 m^3^ d^−1^ until 2030 showed a slight increase compared to those of 2015. The hydraulic head contours at Al-Khrair site appeared smoother and more similar to the hydraulic heads in 2015, while in Al-Shuwaib site, the hydraulic heads were less smooth, with a slight increase in the southeastern part of the site (Fig. 7). These results indicate the capability of both sites to be recharged at the rate of 16,000 m^3^ d^−1^ without significant changes in groundwater flow or excessive head buildup.

**Figure 8:**
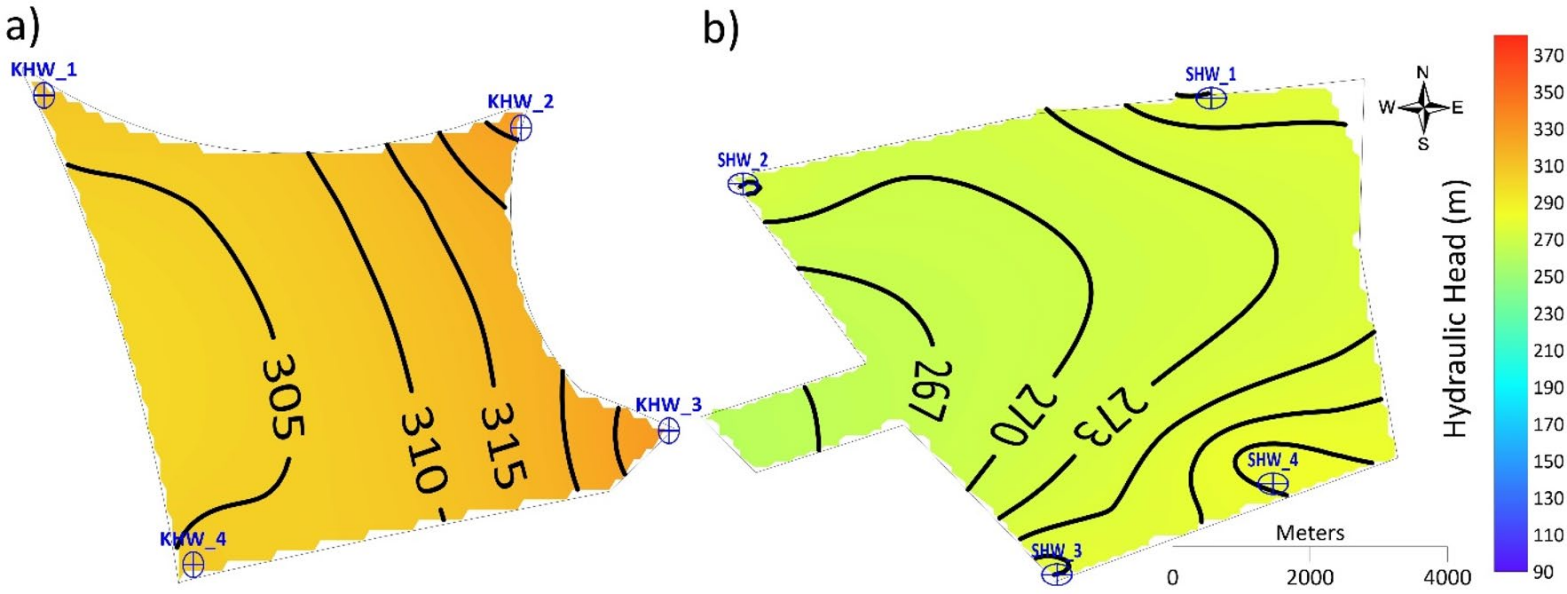
Simulated hydraulic head in 2030 for Scenario 2 at (**a**) Al-Khrair and (**b**) Al-Shuwaib sites.

Two comparisons of a total recharge rate of 32,000 m^3^ d^−1^ were conducted for Scenarios 2 and 3. The hydraulic head values obtained by the four injection wells with a recharge rate of 8,000 m^3^ d^−1^ (Scenario 2) at each site in 2030 are presented (Fig. 8). The hydraulic head values at Al-Khrair site increased at the location of the injection wells, which were situated in the corners of the site, particularly in the east. Changes in groundwater flow and no formation of groundwater mound was observed. For Al-Shuwaib site, the hydraulic head values appeared disturbed in the middle of the site, creating an irregular trend. These results indicate the capability of Al-Khrair site to be recharged by a total of 32,000 m^3^ d^−1^ without significant changes in groundwater flow or excessive head buildup. Although, Al-Shuwaib site was capable of being recharged with the same quantity, it is less suitable compared to Al-Khrair site.

**Figure 9:**
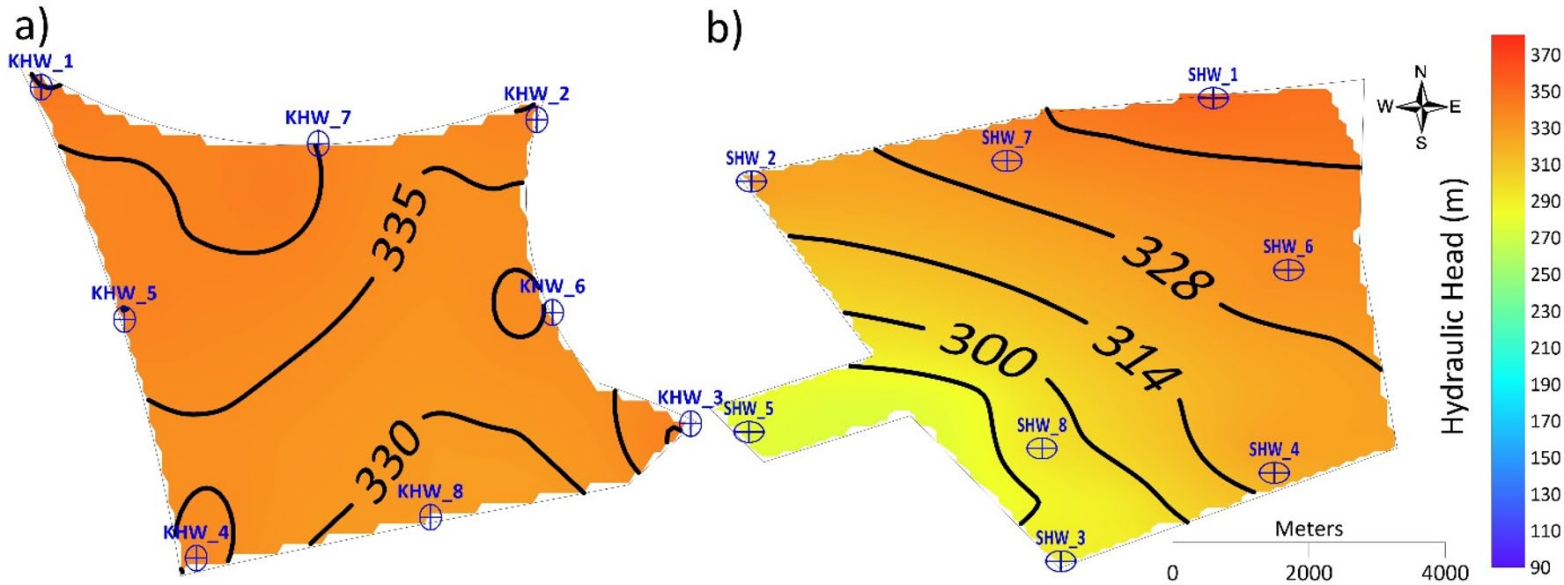
Simulated hydraulic head in 2030 for Scenario 3 at (**a**) Al-Khrair and (**b**) Al-Shuwaib sites.

The hydraulic head obtained from the eight injection wells with a recharge rate of 4,000 m^3^ d^−1^ at each site (Scenario 3) in 2030 is shown in Fig. 9. The hydraulic head at AL-Khrair site in 2030 shows a groundwater mound near injection wells KHW-01 and KHW-07 in the northwestern part of the site. However, the observed hydraulic head did not exceed the ground level. At Al-Shuwaib site, the hydraulic head increased noticeably and exceeded the groundwater level. Therefore, this total recharge rate of 32,000 m^3^ d^−1^ is feasible at Al-Shuwaib site until 2020, without excessive head buildup.

Thus, a total recharge rate of 32,000 m^3^ d^−1^ has less influence on the hydraulic head and groundwater flow when using four injection wells rather than eight injection wells, as the possible interference is less. Both sites can be recharged at the total recharge rate of 32,000 m^3^ d^−1^ by using four injection wells; however, eight injection wells are more suitable for Al-Khrair site.

**Figure 10:**
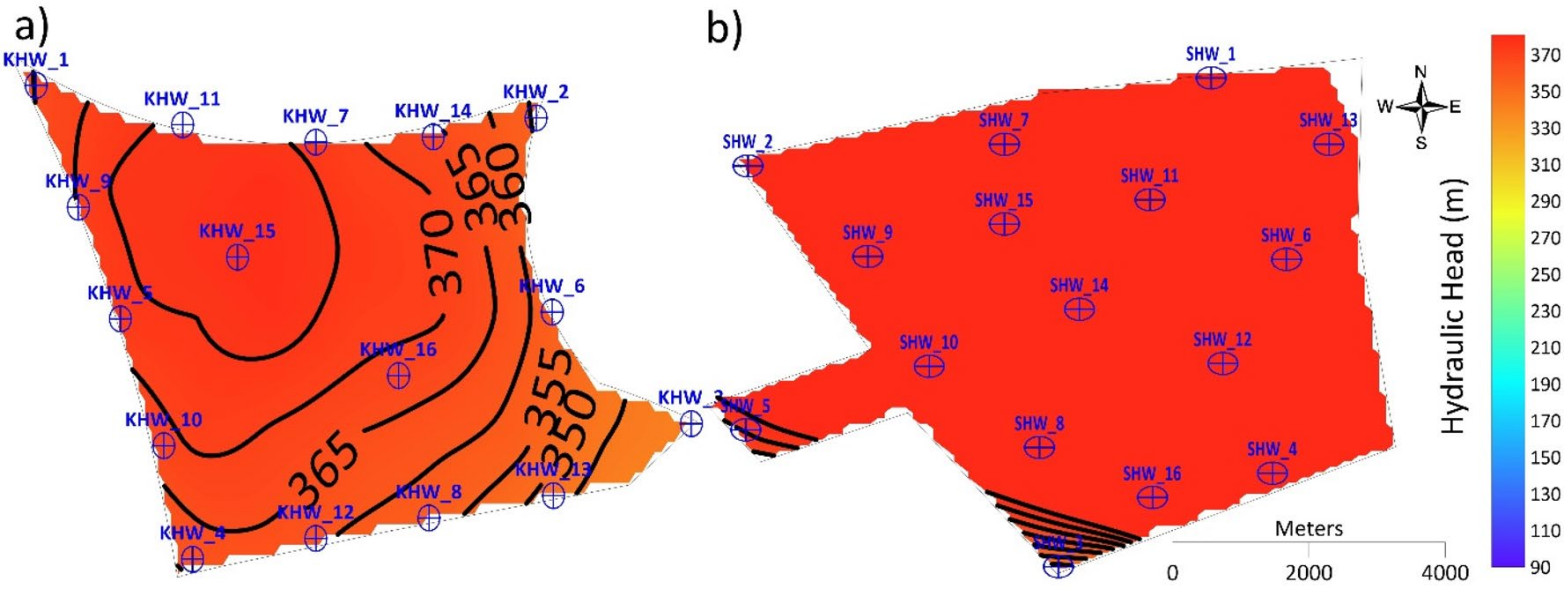
Simulated hydraulic head in 2030 for Scenario 5 at (**a**) Al-Khrair and (**b**) Al-Shuwaib sites.

The hydraulic head obtained from the 16 injection wells with a recharge rate of 4,000 m^3^ d^−1^ at each well in 2030 is shown in Fig. 10. The hydraulic head at Al-Khrair site in 2030 shows a large groundwater mound formed around injection wells KHW-11 and KHW-15 in the northwestern part of the site. At Al-Shuwaib site, the hydraulic head increased significantly with excessive head buildup. This total recharge amount was possible in Al-Shuwaib site until 2015, without excessive head buildup.

**Figure 11:**
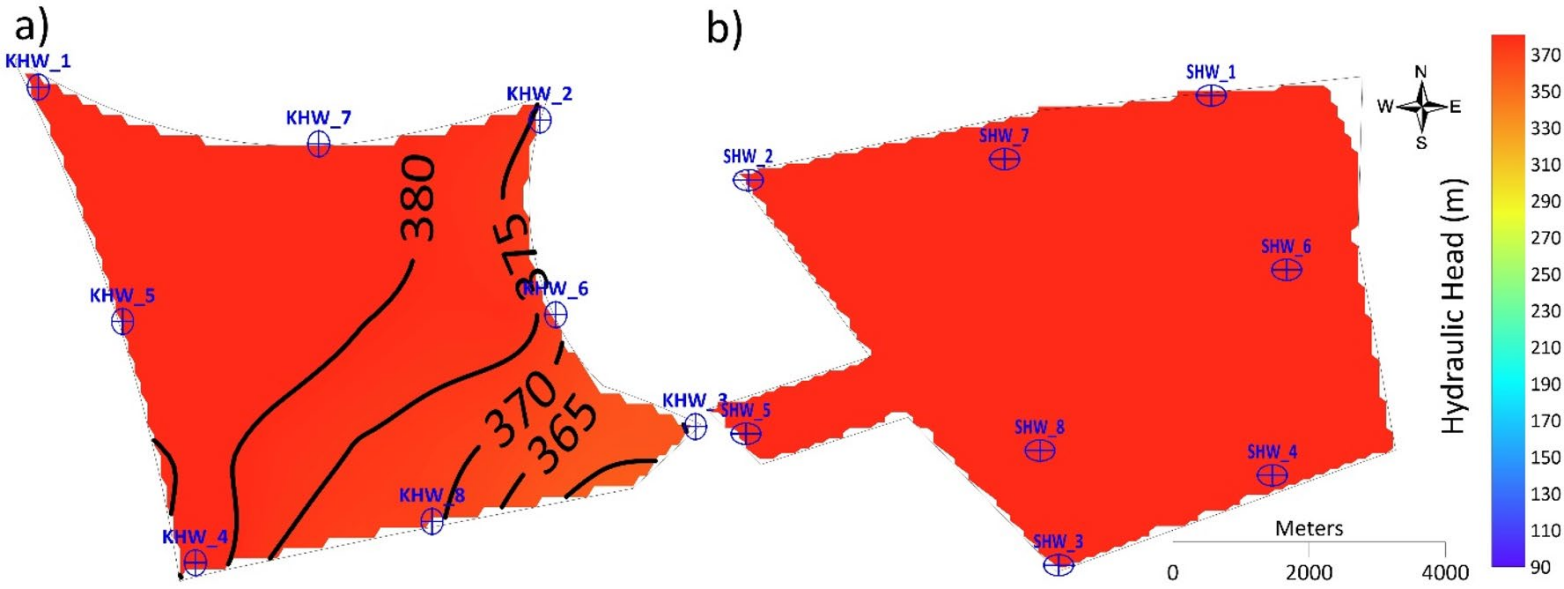
Simulated hydraulic head in 2030 for Scenario 4 at (**a**) Al-Khrair and (**b**) Al-Shuwaib sites.

The hydraulic head obtained from the eight injection wells with a recharge rate of 8,000 m^3^ d^−1^ at each site in 2030 is shown in Fig. 11. The hydraulic head at Al-Khrair site in 2030 shows a large groundwater mound formed near injection wells KHW-01 and KHW-07 in the northwestern part of the site, with hydraulic heads exceeding the ground level after 2020. At Al-Shuwaib site, the hydraulic head increased significantly with excessive head buildup. This total recharge amount was possible in Al-Shuwaib site until 2015, without excessive head buildup.

A summary of recharge rates with possible implementation options at the sites is listed in Table 4.

**Table 4:** Summary of recharge rates with possible implementation options in the sites.

Number of wells	Recharge rate m^3^ d^−1^	Total recharge rate m^3^ d^−1^	Al-Khrair site	Al-Shuwaib site
16	1000	16,000	Applicable until 2030	Applicable until 2030
8	4000	32,000		
4	8000	32,000		Applicable until 2020
8	8000	32,000	Applicable until 2020	Applicable until 2015
16	4000	64,000		

### Comparison of selected sites with potential ASR sites

A study in Washington, USA for estimating the potential local and regional ASR suitability at 284 water wells was conducted based on a site scoring system using hydrogeological, operational, and regulatory components^58^. The ASR suitability method was used to assess the response of groundwater to water storage based on predesigned injection rates ranging from 4,320 m^3^ d^−1^ to 43,200 m^3^ d^−1^. Results revealed that 33 wells had the potential to accommodate an injection rate of 43,200 m^3^ d^−1^. This injection rate is 48% less than the maximum injection rate (64,000 m^3^ d^−1^) simulated at Al-Khrair and Al-Shuwaib sites, indicating their potential for high capacity of strategic water storage.

Al-Khrair and Al-Shuwaib sites achieved high scores in the final ASR suitability map^43,46^, which is based on two criteria groups related to hydrological and complementary components. These criteria groups represent the performance of the local aquifer and the feasibility of the ASR system. The results presented here agree well with similar ASR suitability approaches implemented in several studies^44,45,47^. It has been demonstrated that the main factors that influence the potential of groundwater recharge zones in Flinders Ranges, South Australia are lithology, gentle slope/gradient, and alluvial deposits^59^. In addition, a study in the Prachinburi province of Thailand indicated that the presence of unconsolidated material enhances the potential for groundwater recharge due to its high porosity and permeability^60^.

Several authors have studied the suitability of the groundwater aquifer in Al Ain City over the last few years. Each author used different criteria to evaluate future ASR projects. For instance, EAD^13^ used hydrogeological criteria, such as the thickness of the aquifer, thickness of the unsaturated zone, aquifer confinement, quality of native groundwater, aquifer transmissivity and storativity, hydraulic gradient, and the presence of a third party using the aquifer. In addition, there are few other criteria, such as distance to the closest border or coastline, infrastructure, environmental aspects, and land development.

**Figure 12:**
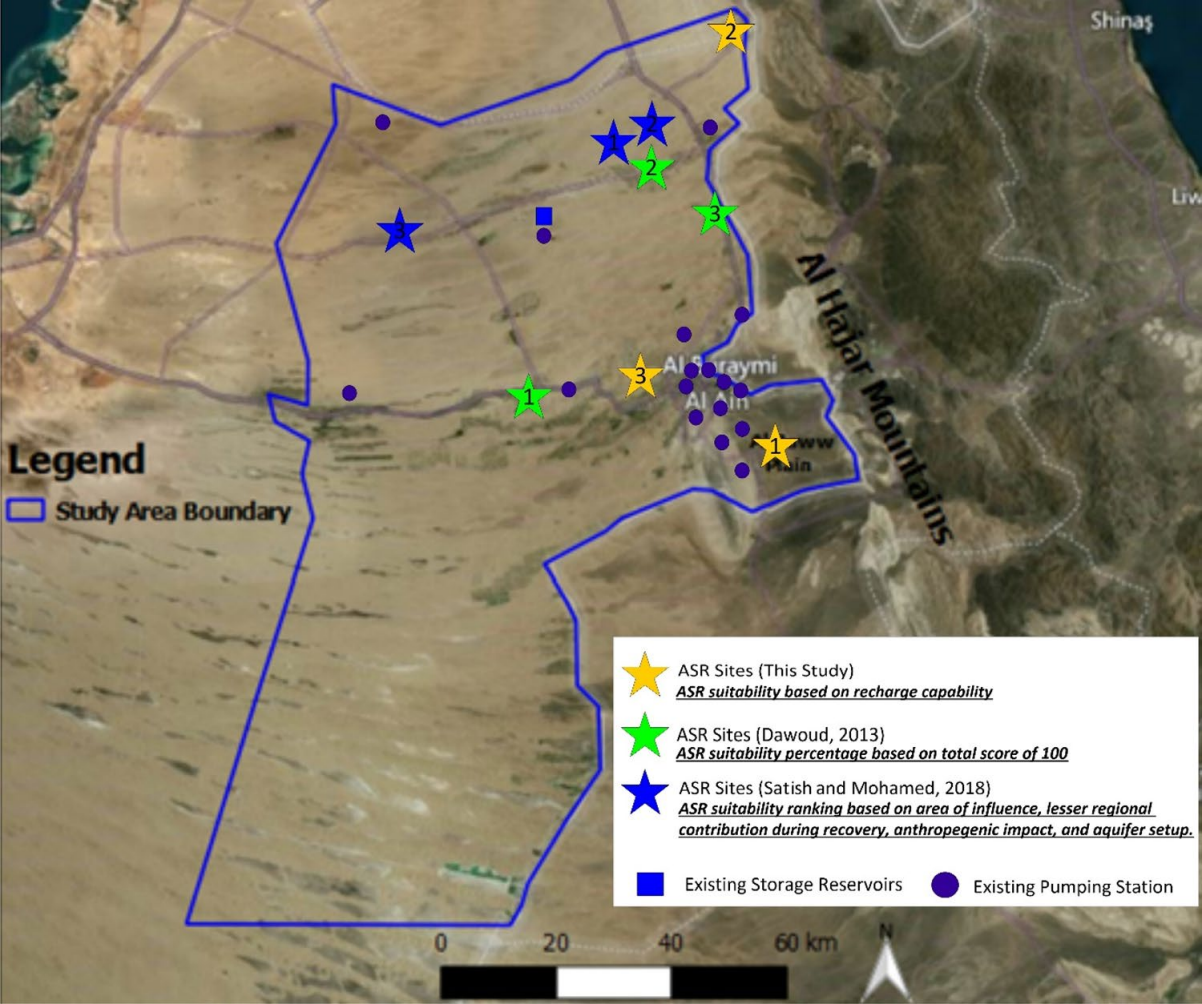
Suitable sites for the aquifer storage and recovery system for other studies and those identified in this study.

Sathish and Mohamed^25^ studied the possibility of ASR occurring in the Emirate of Abu Dhabi. In this investigation, hydrogeological criteria, as well as the performance of each site in terms of rate of injection, total volume of injection, recovery efficiency, radius of influence, and type of cone were used to find the most suitable location in the Emirate of Abu Dhabi, including the study area. The suitable sites in the Al Ain region for an ASR system, based on this study and previous studies^25,61^, according to their suitability ranking are shown in Fig. 12.

The suitable sites identified in this study (yellow stars # 1 and 2) are in the east of the study area. A third site (yellow star # 3), ranked third in^43^, is added in Fig. 12 to compare among the best three sites selected in three different studies using different selection criteria. In the last decade, the groundwater levels at Al-Khrair and Al-Shuwaib sites were located within a stable zone according to the groundwater level map developed by EAD^62^. Al-Shuwaib site is located near Sites 1 and 2 of those identified by Sathish and Mohamed^25^ and sites 2 and 3 of those identified by Dawoud^61^. Sites 1 and 2 from Sathish and Mohamed^25^ are located at the Sweihan site, whereas Site 1 from Dawoud^61^ is located at Al-Saad site. According to the ASR site selection process implemented on the 20 discrete sites within the study area^43^, the Sweihan site shows 73% of suitability based on ASR, whereas Al-Saad site has 70% suitability.

The Sweihan site was not selected here due to the relatively high salinity of groundwater in the aquifer and the continuous withdrawal of groundwater for agricultural activities^63,64^. Al-Saad site was not selected for the same reasons of the Sweihan site^49^. These sites were previously considered suitable for ASR systems based on the available data and considering different selection criteria including the presence of surface storage tanks/water facilities located the selected sites. In addition, most of the selected sites were located east of the study area, probably because the aquifer was characterized with good quality of water. In general, the selected sites in this study are different from other sites selected in previous studies; as shown in Fig. 12. This shows that the used criteria and the accuracy of used data could affect site selection process. The weighting factors used in the selection criteria is subjective to authors assessment of the important factors to be considered. Modeling, therefore, is a crucial step to confirm the feasibility of a selected site for a successful implementation of an ASR system.  

### Ranking of selected sites

The sites studied for the ASR project are in Al-Khrair and Al-Shuwaib areas. Each site has its own hydrogeological characteristics and recharge capacity limits according to the simulated hydraulic heads during 2013–2030. An additional assessment of Al-Khrair and Al-Shuwaib sites was implemented to identify the best site for the ASR system, which can be recharged with a large quantity of water without changing groundwater flow or causing excessive head buildup. According to borehole data, the groundwater table for Al-Khrair site was encountered at a depth of approximately 40 m below ground surface, whereas for Al-Shuwaib site, the groundwater table was encountered at a depth of approximately 20 m below ground surface. These depths are expected to be shallower after water recharge from the injection wells. Therefore, it is preferable to have a deeper groundwater table, as there is less chance of contamination in the aquifer from sources on the ground surface^35,65^. In addition, Al-Khrair site is located to the east of the study area on the Al-Jaww plain, which is close to the border of the Sultanate of Oman, approximately 12 km away. Al-Shuwaib site is located 8 km north of the border of the Sultanate of Oman. Furthermore, several pumping stations are in the vicinity of Al-Khrair site, while for Al-Shuwaib site, the only nearby pumping station is in Al-Hayer area 19 km away. Geophysical investigation using the gravity method was carried out in the Al-Jaww plain^66^, wherein the proposed Al-Khrair site is located, revealing a major syncline (bowl-shaped geological formation) because of a strong negative anomaly (low gravity) as well as a series of anticlines. This confirms the presence of the prerequisite stated by Maliva et al.^67^ for achieving a useful storage of water, wherein the ASR system must have an aquifer with lateral boundaries, which act as the wall of the tank as long as there is no leakage from the storage zone^67^.

Based on the above, Al-Khrair site is more suitable than Al-Shuwaib site. Al-Khrair site has good potential for storage of water as there is a bowl-shaped structure (syncline). In addition, there are similarities between Al-Khrair site and the currently implemented ASR project in the Liwa area of the Emirate of Abu Dhabi in terms of the presence of natural groundwater with low salinity, permeable geological layers, adequate aquifer thickness, and sufficient depth to the groundwater table^68^.

## Conclusions

The aim of this study was to examine suitable sites that can be recharged with surplus desalinated water to enhance the depleted aquifer in the eastern district of Abu Dhabi for the purpose of use as a strategic water reserve for recovery with high efficiency at later periods to meet the ensuring high demand. The examined sites were selected using an ASR suitability map that was developed based on two criteria groups’, which are directly related to the performance and feasibility of the ASR system. Aquifer recharge at Al-Khrair and Al-Shuwaib sites was simulated using the Visual MODFLOW Flex 2015.1. Five scenarios of water recharge were simulated, including 16,000 m^3^ d^−1^, 32,000 m^3^ d^−1^, and 64,000 m^3^ d^−1^. The suitability of locating the ASR system was analyzed by comparing Al-Shuwaib and Al-Khrair sites. The site suitability assessment was performed using closely spaced injection wells (approximately 1,200 m); however, because of the excessive buildup of the hydraulic head, a new distribution of injection wells was assigned to the sites with a wider spacing (> 1200 m) to avoid the overlap and interference caused by the developed groundwater mounds at each well. The new distribution of injection wells was further assessed for Al-Khrair and Al-Shuwaib sites. Assessment results suggest that Al-Khrair site can be recharged with 64,000 m^3^ d^−1^ for seven years continuously before changing the behavior of groundwater flow and developing excessive head buildup. In the case of the Al-Shuwaib site, it is possible to be recharged at the rate of 64,000 m^3^ d^−1^ for only two years. This groundwater model provides a better understanding of the capabilities and constraints of the potential ASR site using different injection rates. Results of this study provide cost-effective solutions for the sustainability of groundwater in Al Ain region.

## Data availibility

The datasets used and/or analysed during the current study are available from the corresponding author on reasonable request.

## Supplementary Information

Supplementary Information.
