# Supplementary material for: Modeling aquifer storage and recovery in the eastern district of the United Arab Emirates using MODFLOW
Source: Sci Rep. 2022 Oct 20;12:17537. doi: 10.1038/s41598-022-20470-7 (PMC9584962; doi:10.1038/s41598-022-20470-7)
Supplement: Supplementary file 1 — Supplementary Information. [file 41598_2022_20470_MOESM1_ESM.docx]

Appendix A

**Figure A.1.** Calculated versus observed calibration chart for a) 2014, b) 2015, and c) 2016 ACES observation wells

**Figure A.1(cont.).** Calculated versus observed calibration chart for a) 2014, b) 2015, and c) 2016 ACES observation wells
